# An Intervention With Dance and Yoga for Girls With Functional Abdominal Pain Disorders (Just in TIME): Protocol for a Randomized Controlled Trial

**DOI:** 10.2196/19748

**Published:** 2020-12-15

**Authors:** Anna Philipson, Stefan Särnblad, Lars Ekstav, Mats Eriksson, Ulrika L Fagerberg, Margareta Möller, Evalotte Mörelius, Anna Duberg

**Affiliations:** 1 University Health Care Research Center Faculty of Medicine and Health Örebro University Örebro Sweden; 2 Department of Paediatrics Örebro University Hospital Örebro Sweden; 3 Faculty of Medicine and Health School of Medical Sciences Örebro University Örebro Sweden; 4 Faculty of Medicine and Health School of Health Sciences Örebro University Örebro Sweden; 5 Centre for Clinical Research Västmanland Hospital Västerås Uppsala University Uppsala Sweden; 6 Department of Women's and Children's Health Karolinska Institutet Stockholm Sweden; 7 Department of Health, Medicine and Caring Sciences Linköping University Linköping Sweden; 8 School of Nursing and Midwifery Edith Cowan University Joondalup Australia

**Keywords:** dance, functional abdominal pain, functional abdominal pain disorders, irritable bowel syndrome, physical activity, randomized trial, study protocol, yoga

## Abstract

**Background:**

Functional abdominal pain disorders (FAPDs) affect many children worldwide, predominantly girls, and cause considerable long-term negative consequences for individuals and society. Evidence-based and cost-effective treatments are therefore strongly needed. Physical activity has shown promising effects in the practical management of FAPDs. Dance and yoga are both popular activities that have been shown to provide significant psychological and pain-related benefits with minimal risk. The activities complement each other, in that dance involves dynamic, rhythmic physical activity, while yoga enhances relaxation and focus.

**Objective:**

This study aims to evaluate the effects of a dance and yoga intervention among girls aged 9 to 13 years with FAPDs.

**Methods:**

The study is a prospective randomized controlled trial among girls aged 9 to 13 years with functional abdominal pain, irritable bowel syndrome, or both. The target sample size was 150 girls randomized into 2 arms: an intervention arm that receives dance and yoga sessions twice weekly for 8 months and a control arm that receives standard care. Outcomes will be measured at baseline and after 4, 8, 12, and 24 months, and long-term follow-up will be conducted 5 years from baseline. Questionnaires, interviews, and biomarker measures, such as cortisol in saliva and fecal microbiota, will be used. The primary outcome is the proportion of girls in each group with reduced pain, as measured by the faces pain scale-revised in a pain diary, immediately after the intervention. Secondary outcomes are gastrointestinal symptoms, general health, mental health, stress, and physical activity. The study also includes qualitative evaluations and health economic analyses. This study was approved by the Regional Ethical Review Board in Uppsala (No. 2016/082 1-2).

**Results:**

Data collection began in October 2016. The intervention has been performed in 3 periods from 2016 through 2019. The final 5-year follow-up is anticipated to be completed by fall 2023.

**Conclusions:**

Cost-effective and easily accessible interventions are warranted to reduce the negative consequences arising from FAPDs in young girls. Physical activity is an effective strategy, but intervention studies are needed to better understand what types of activities facilitate regular participation in this target group. The Just in TIME (Try, Identify, Move, and Enjoy) study will provide insights regarding the effectiveness of dance and yoga and is anticipated to contribute to the challenging work of reducing the burden of FAPDs for young girls.

**Trial Registration:**

ClinicalTrials.gov (NCT02920268); https://clinicaltrials.gov/ct2/show/NCT02920268

**International Registered Report Identifier (IRRID):**

DERR1-10.2196/19748

## Introduction

Functional abdominal pain disorders (FAPDs) [1,2] affect 13.5% of school-aged children worldwide [3] and are associated with low quality of life [4]. The prevalence is substantially higher among girls (15.9%) than boys (11.5%) [3]. The negative consequences of FAPDs, such as absence from school [4,5], depression [4,6], and high consumption of medical care [7], pose a considerable burden on children and their families. It is common for pain to be sustained throughout the school years [8-11]. Furthermore, somatic symptoms in childhood predict severe mental illness in adulthood [12]. Specifically, FAPDs in childhood can lead to long-term vulnerability to anxiety later in life, even if abdominal pain resolves [13]. Children need early treatment and preventive strategies to address these symptoms [14] regardless of the presence of co-occurring mental health disorders [12].

For almost 90% of children with chronic abdominal pain, no explanatory organic cause can be identified [15], and psychosocial factors contribute to the development and maintenance of the disease [16]. The predisposing factors and pathophysiological mechanisms of FAPDs include visceral hypersensitivity, altered gastrointestinal motility, and changes in intestinal microbiota, as well as stressful events [3,17], mental health issues, and negative experiences, such as bullying [18]. These children are likely to have poor coping strategies for stressful situations [19]. Thus, interventions and therapeutic modalities that address stress reduction are frequently discussed [3].

There is currently no convincing evidence for treatment or symptom relief with pharmaceuticals [17,20-22] or dietary treatment [23]. Nonpharmacological interventions, such as hypnotherapy and different types of cognitive behavioral therapy (CBT), have shown both short- [17,19,24] and long-term pain relief for children with FAPDs [24]. However, many of these interventions are time-consuming or require specially educated staff [17]. Effective strategies for managing FAPDs in children include reducing both parent and child concerns about the seriousness of the condition and, instead of striving for total pain relief, reducing the disability associated with pain [25] and improving the quality of life for the child [25-27].

Physical activity has been shown to be effective in the practical management of FAPDs [18] by distracting from the pain and improving function. Unfortunately, levels of physical activity among young girls is alarmingly low, which calls for action [28]. Dance is a popular physical activity among young girls [29] that can positively influence physical health outcomes [30], motor skills [31], and psychological well-being [32,33]. Through the expressive, creative, and aesthetic aspects of physical activity, dance holds potential to enhance body awareness and improve poor body image, which in turn strengthens self-esteem [34,35]. In a social context, dance is a cost-effective intervention [36] that can reduce somatic and emotional stress-related problems [37], increase self-rated health [38], and enhance self-esteem [39] and feelings of enjoyment and energy [40-42]. For girls aged 8 to 12 years, multicomponent interventions including dance can lead to improvements in psychological well-being, perceived self-efficacy, and physical self-confidence [43]. Although more research is needed, dance has also been proven to help decrease pain, both for young people [44] and adult women [45-47].

Yoga is a psychophysiological practice with a focus on posture, controlled breathing, and attention [48]. For children, yoga has been shown to improve focus and emotional regulation [49] and to effectively reduce anxiety [50,51] and depression [52]. Yoga has also gained popularity in the treatment of pediatric FAPDs [27,53-55]; studies have shown reductions in abdominal pain frequency [56], pain intensity [56,57], and school absence [57]. For children with irritable bowel syndrome (IBS), yoga has been shown to improve quality of life and physical functioning [58,59] and reduce IBS symptoms [58-60], but more research is needed [20].

A combination of physical and mental training has been shown to be beneficial in reducing stress and increasing quality of life [61]. Coupled together, studies indicate a potentially higher effectiveness than one modality alone [61,62]. Choreographed dance routines to popular music combined with calm breathing meditation has been shown to decrease symptoms of depression and anxiety for homeless women [63].

Dance and yoga have been acknowledged in recent research as pain management for young girls [64] and are both noncompetitive activities that can appeal to girls, which in turn can positively impact participation rates [65]. Dance and yoga can complement each other because dance involves dynamic, rhythmic physical activity, while yoga enhances relaxation and focus [66]. Both dance and yoga focus on body awareness, which has been shown to provide significant psychological and pain-related benefits with minimal risk [67,68], and can also meet young people’s desire to self-manage symptoms with accessible treatment options and to take a more active role in their own care [69]. However, more controlled trials of nonpharmacological interventions are warranted [70], especially for stress-modulated conditions in youth [67]. The novelty of the intervention type and the vulnerability of the target group call for extensive investigation, which we aim to accomplish with a longitudinal randomized design and different methodologies. To our knowledge, dance and yoga for children with FAPDs have not previously been studied.

The overall aim of the study is to evaluate the effects of a dance and yoga intervention among girls aged 9 to 13 years with FAPDs.

## Methods

### Approval and Registration

The study was approved by the Regional Ethical Review Board in Uppsala (No. 2016/082 1-2) and is registered on ClinicalTrials.gov (NCT02920268). Any protocol modifications will be communicated to relevant parties.

### Study Design

**Figure 1:**
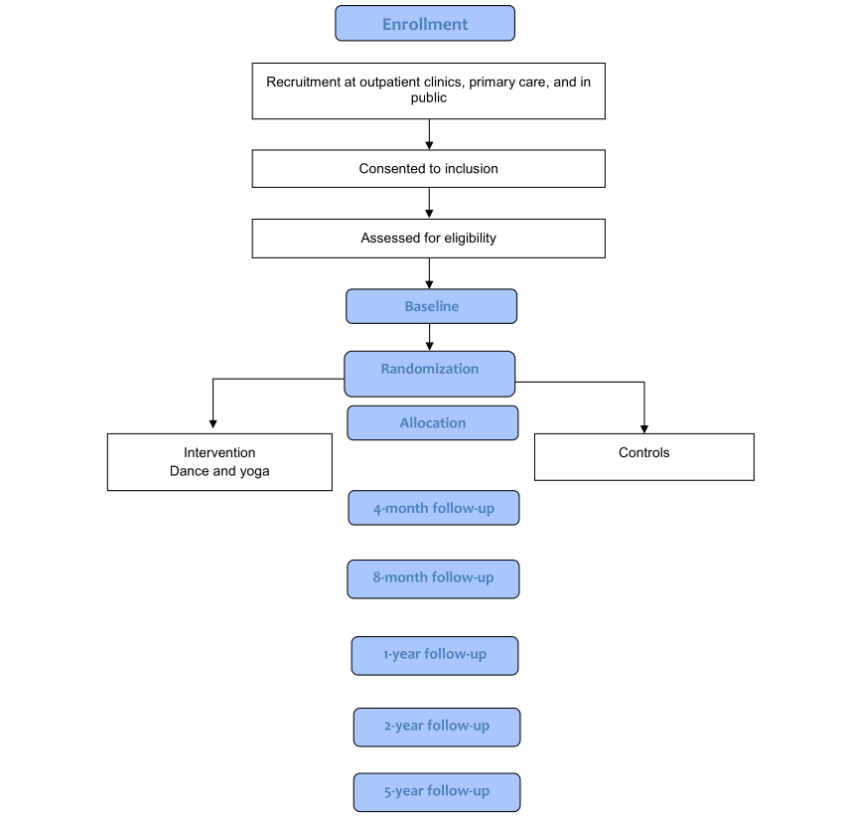
Flowchart of the protocol.

This study, called Just in TIME (Try, Identify, Move, and Enjoy), started in 2016 when the first intake was performed and will end in 2023. The study is a prospective randomized controlled trial with 2 parallel groups—an intervention group and a control group—of girls aged 9 to 13 years with functional abdominal pain, IBS, or both. The intervention consists of dance and yoga sessions 2 times a week for 8 months. The control group receives standard care as school health care or primary care. The outcomes are measured at baseline and after 4, 8, 12, and 24 months. A long-term follow-up will be performed 5 years from baseline (Figure 1). At all follow-ups, data are collected for both the intervention and control groups. The trial is being conducted in 2 cities in Sweden.

The study follows the standard methodology of intervention research. This protocol is conducted according to the SPIRIT (Standard Protocol Items: Recommendations for Interventional Trials) guidelines [71], and the description of the intervention follows the TIDieR (Template for Intervention Description and Replication) checklist and guide [72]. The study will be reported according to the CONSORT (Consolidated Standards of Reporting Trials) guidelines [73], COREQ (Consolidated Criteria for Reporting Qualitative Research) [74], and CHEERS (Consolidated Health Economic Evaluation Reporting Standards) [75].

### Study Population

According to international guidelines (Rome IV), FAPDs include functional dyspepsia, IBS, abdominal migraine, and functional abdominal pain not otherwise specified with persisting symptoms 2 months prior to diagnosis [1]. The previous term for these diagnoses according to Rome III was abdominal pain-related functional gastrointestinal disorders (FGIDs) [2].

The Rome III criteria provided the current guidelines during the time of the first study inclusion. The study population includes girls diagnosed with functional abdominal pain or IBS according to the Rome III criteria [2] and with persistent pain after examination at the pediatric center. The girls were aged 9 to 12 years during the first year for intake and 9 to 13 years during the second and third intake years.

#### Inclusion Criteria

Inclusion criteria followed the diagnostic criteria for childhood functional abdominal pain in Rome III [2]: (1) episodic or continuous abdominal pain at least once per week for at least 2 months before diagnosis; (2) insufficient criteria for other FGIDs; and (3) no evidence of an inflammatory, anatomic, metabolic, or neoplastic process that explains the patient’s symptoms.

The following criteria were tested to exclude other illnesses: (1) physical examination including normal growth pattern and (2) laboratory screening, including serological screening with immunoglobulin A antibodies against type 2 (tissue) transglutaminase (TGA-IgA), complete blood cell count, erythrocyte sedimentation rate or C-reactive protein, and urine analysis. A fecal calprotectin test was also included if the girl reported symptoms such as diarrhea.

Diagnostic criteria for IBS in Rome III [2] were used. First, at least once per week for at least 2 months before diagnosis, the patient must have had abdominal discomfort or pain associated with 2 or more of the following at least 25% of the time: (1) improved with defecation, (2) onset associated with a change in the frequency of stool, or (3) onset associated with a change in the form (appearance) of stool. Second, there must be no evidence of an inflammatory, anatomic, metabolic, or neoplastic process that explains the patient’s symptoms.

The following criteria were tested to exclude other illnesses: (1) physical examination including normal growth pattern and (2) laboratory screening including serological screening with TGA-IgA, complete blood cell count, erythrocyte sedimentation rate or C-reactive protein, and urine analysis. A fecal calprotectin test was also included if the girl reported symptoms such as diarrhea.

Persistent pain after examination at the pediatric center was measured with a pain diary at baseline. Girls who reported one or more episodes of pain with a pain score of 4 or higher on the faces pain scale-revised (FPS-r) [76,77] during a full week were eligible for the study.

#### Exclusion Criteria

The exclusion criteria for this trial were (1) contemporaneous celiac or inflammatory bowel disease; (2) difficulty following oral instructions, such as hearing impairment, mental retardation, or language difficulties; (3) simultaneous treatment with CBT; and (4) severe psychological symptoms for which other treatment is needed.

#### Recruitment

Participants were recruited from outpatient clinics, primary health care services, and the public.

We recruited participants from the outpatient clinics of the pediatric departments at the university hospitals in Örebro, Karlskoga, and Lindesberg, Sweden. In the second recruitment year, participants were also recruited from the pediatric outpatient clinic in Västerås, Sweden. All girls aged 9 to 13 years (9 to 12 years during the first year of the study) who had visited one of the outpatient clinics during the previous 2 years because of abdominal pain and had received a diagnosis of IBS, functional abdominal pain, constipation, or abdominal pain received an information letter asking whether they wanted to join the study. All participants were found via local diagnosis registries at the included hospitals. After written consent was received from the legal guardians, the diagnosis according to the inclusion criteria was verified in the medical records or by examination by a pediatrician.

In addition, we recruited girls from primary health care services in the region of Örebro and Västerås. Information letters were delivered to potential participants and their legal guardians in primary care, the counselling unit, and school health services. Interested girls and their legal guardians contacted the research team, who distributed an information letter and the consent form. After written consent was obtained, the research team booked an appointment for an examination at the pediatric clinic (Örebro) or research clinic (Västerås).

Information about the study project was provided by several information channels, such as social media, ordinary media, and websites. Interested girls and their legal guardians contacted the research team, who distributed an information letter and the consent form. After written consent was obtained, the research team booked an appointment for an examination at the pediatric clinic (Örebro) or research clinic (Västerås).

Girls and their legal guardians who consented to the study and were eligible for the study according to the inclusion criteria completed the baseline measurement.

#### Randomization

When the baseline measurement was completed and the eligibility check was accomplished, the sample was randomized into an intervention group and a control group. Randomization was performed by an external statistician using minimization based on pain intensity and age at baseline [78].

#### Sample Size

The total number of required study participants was calculated for the primary outcome (decreased maximal pain after 8 months). The calculation was based on several assumptions. First, we considered the expected proportion of participants who decreased their maximal pain after 8 months in the intervention group. We estimated that 30% of the intervention group would decrease their maximal pain after 8 months based on previous research with CBT, in which approximately 50% of participants were pain free immediately after the intervention [79]. Second, we calculated the expected proportion of participants whose maximal pain would decrease after 8 months in the control group. In previous CBT studies, approximately 25% of control participants reported being pain free when the intervention ended [79]. Since only girls with a long duration of abdominal pain were included in the present study, we estimated that the placebo effect would be smaller and that 10% of the girls in the control group would report decreased maximal pain at the 8-month follow-up. Third, dropout is a common problem that can bias outcomes. A 20% to 50% dropout rate is commonly reported in studies of participants with long-lasting pain [80]. In the present study, a 20% dropout rate was estimated.

With a power of 80% and a significance level of .05, we calculated the sample size to be N=150 (75 intervention + 75 control participants), including a 20% dropout. To decrease bias, a minimum of 50 participants in each group is recommended in trials studying intervention effects on pain, according to previous literature [80].

### Intervention

The description of the intervention follows the TIDieR checklist and guide [72].

#### Name and Rationale

The intervention is called Just in TIME and addresses the importance of early intervention for this target group. The word “TIME” stands for “Try, Identify, Move, and Enjoy,” which also characterizes the key aspects of the intervention, which aims to decrease FAPDs among 9- to 13-year-old girls through dance and yoga. Essential elements of the intervention included a focus on enjoyment, socialization, and playful creativity in an undemanding environment. We chose a combination of dance and yoga because of experiences from an earlier study [38] that targeted adolescent girls with internalizing problems and evaluated a dance intervention. The study participants appreciated the intervention and rated it to be a positive experience [38], but at the end of the intervention period, they started to request yoga as an add-on at the end of the sessions. They believed that it would be a valuable closure to the session to help them tune in to the relaxation. These requests were a form of evaluation that the research team took into account when designing the current study.

#### Materials

Informational material was distributed during the training of the intervention providers. It consisted of written course materials and visually recorded practical choreography and sequences, all provided by the intervention coordinator.

#### Procedures

The intervention was performed as a group activity twice a week after school hours during an 8-month period. The duration of the class was 60 minutes, comprising 30 minutes of dance practice, 25 minutes of yoga including relaxation, and 5 minutes of short reflection (Table 1). Throughout the intervention period, the participants were encouraged to practice their favorite dances, yoga poses, or relaxation techniques at home if they wanted to. Both centers followed the same routine.

**Table 1:** The 60-minute Just in TIME dance and yoga class.

Minutes	Section	Description
10	Dance: warm-up	The warm-up section aimed to get the participating girls to be active and take part in the social cohesion. It included up-tempo music with prominent drum beats and captivating rhythm. Expansive, easy, accessible movements activated large muscle groups.
20	Dance	The dance choreography section included mostly structured dance as a group under the guidance of an instructor but also included improvisation and playful exploration of movement. The focus was on enjoyment and socialization rather than performance. The intention was to offer an opportunity to experience one’s own body in a positive way with popular music in an undemanding and supportive atmosphere as well as to increase heart rate with moderate-to-vigorous physical activity. Month 1-2: Focus on experiencing joy of movement, feeling safe, and getting to know each other. Dance style: show jazz. Month 3: Focus on expansive movements, “claiming space,” determination, and integrity. Dance style: show jazz and street dance. Month 4: Focus on body awareness, slow movements, and grace. Dance style: jazz and contemporary dance. Month 5-6: Focus on increasing energy and working together as a group in the choreography. Dance style: show jazz. Month 7-8: Focus on enjoyment and meeting variations in the dance movements (high-low, firm-soft, and expansive-small variations). Dance style: show jazz and floor work.
20	Yoga	The yoga section focused on playful movements, such as creative yoga storytelling combined with asanas (body poses), a focus on breathing, and attention. Asanas were performed individually or in pairs and, when appropriate, together as a group. Month 1-2: *balasana* (child’s pose), *marjaryasana* (cat pose) and *bitilasana* (cow pose), *parsva sukhasana* (seated side bend pose), *uttanasana* (standing forward bend), *upavistha bitilasana marjaryasana* (seated cat-cow pose), *jathara parivrtti* (revolved abdomen twist pose) Month 3: *parivritta sukhasana* (sitting twist pose), *parsvaparvatasana in tadasana* (standing side bend), *uttanasana* (standing forward bend), *sufi grind* (seated torso circles), *paranamuktasana* (knees to chest), *jathara parivrtti* (revolved abdomen twist pose) Month 4: *virabhadrasana* II (warrior II), *ardha chandrasana* (half-moon pose), *ardha setubandhasana* (half-bridge pose), *adho mukha sukhasana* (easy pose forward bend), *upavistha bitilasana marjaryasana* (seated cat-cow pose), *jathara parivrtti* (revolved abdomen twist pose) Month 5-6: *vrksasana* (tree pose), *utthita trikonasana* (extended triangle pose), *upavista konasana* (seated wide angle posture), *adhomukha svanasana* (downward-facing dog posture), *balasana* (child’s pose), *paranamuktasana* (knees to chest), *sufi grind* (seated torso circles), *jathara parivrtti* (revolved abdomen twist pose) Month 7-8: extended focus on creative yoga storytelling in group, *phalakasana* (plank pose), *adhomukha svanasana* (downward-facing dog posture), *bhujangasana* (cobra pose), *ardha setubandhasana* (half-bridge pose), *jathara parivrtti* (revolved abdomen twist pose)
5	Relaxation	*Pranayama* (slow deep breathing and attention to the breath), *savasana* (corpse pose), guided relaxation to increase calmness, and lying down with blankets. During the relaxation, a brief massage on the shoulders was offered by the instructors (voluntary).
5	Reflection	Finally, a short voluntary sharing session was held while seated in a circle, highlighting a positive experience during this particular class.

#### Intervention Providers

Each group included 7 to 14 girls and was under the guidance of 2 instructors, one at a time. The instructors had a profession in either health care or pedagogy, had experience working with young people, and were educated in teaching children or adolescents in dance (instructor training) and yoga (ie, registered yoga teacher 200-hour training). Prior to the start of the intervention, all instructors attended a 2-day course administered by the research team. This course consisted of practical instructions from the intervention coordinator about the dance and yoga session according to the standardized program design, including dance choreographies and yoga sequences adjusted to the target group as well as teaching style (nonjudgmental and supporting approach). Lectures about theories and underlying mechanisms and about guidelines and ethical aspects regarding children with FAPDs were also given.

#### Modes of Delivery

Various styles of dance with a focus on enjoyment were performed during the intervention period. The yoga practice consisted of both playful movements (such as creative yoga storytelling) and calm physical postures with a focus on breathing and attention, which were performed individually, in pairs, and as a group. The intervention (further described in Table 1) was developed by the intervention coordinator (author AD). To ensure standardization in program delivery across both centers and between instructors over time, booster sessions were given 3 times during the intervention period (in addition to the initial 2-day course). The participants in the intervention were advised to wear practical soft clothes suitable for the activity, and no special shoes were needed.

#### Locations

The intervention took place in studios located in the center of Örebro and Västerås, Sweden, which were easily accessible by bus or walking. The studios had yoga mats and music equipment.

#### Frequency and Duration

The 8-month period was chosen because it corresponded to 2 school semesters. No classes were held during holidays; thus, 50 dance classes were held over 25 weeks. The practice can be classified as moderate-to-vigorous physical activity.

#### Tailoring and Modification

To address modifications, both dance and yoga movements were introduced in steps to include all participants regardless of previous experience. Alternatives were given when needed. No changes were made in the intervention.

#### Adherence

Adherence to the dance and yoga intervention was noted by the instructors. A strategy to maintain and improve fidelity was to consciously focus on relatedness and provide a feeling of social inclusion, supportiveness, and the chance to meet new friends. To keep the dance and yoga sessions interesting over the time period, a variety of styles and themes were presented, all distributed by the intervention coordinator. The intervention was delivered as planned throughout the 3 intervention years.

### Outcomes

**Figure 2:**
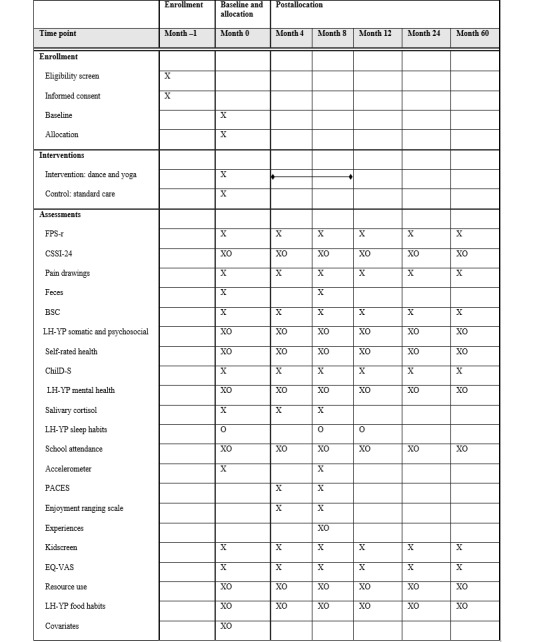
Enrollment, interventions, and assessments. An “X” represents a child report and an “O” represents a legal guardian report. The PACES, enjoyment rating scale, and experiences questions were only asked to the girls in the intervention group. BSC: Bristol stool chart; ChilD-S: Children’s Depression Screener; CSSI-24: Children’s Somatic Symptoms Inventory-24; EQ-VAS: EuroQol visual analog scale; FPS-r: faces pain scale-revised; LH-YP: Life and Health – Young People; PACES: Physical Activity Enjoyment Scale.

This study includes biochemical and physiological measures, questionnaires (for the girls in the study and their legal guardians who answer questions about their daughters as a proxy), and qualitative interviews (with both the girls in the study and their legal guardians) (Figure 2). The questionnaire sessions are performed at the research centers, and the project team provides assistance. To retain as many participants as possible, reminder mail and emails are sent to participants. Notes about participants who discontinue or deviate from the study are made.

#### Primary Outcome Measure

The primary outcome is the proportion of girls in each group who have reduced maximal pain, measured immediately after the intervention (8-month follow-up) with the FPS-r [76,77] in a pain diary in which the girls register their abdominal pain 3 times a day for 1 week. The psychometric features for the FPS-r are reported to be good [77].

#### Secondary Outcome Measures

##### Abdominal Pain and Gastrointestinal Issues

Abdominal pain is measured with the pain diaries with the FPS-r [76,77].

Abdominal pain is also measured in the questionnaires with a subscale in the Children’s Somatic Symptoms Inventory-24 (CSSI-24) (formerly known as the Children’s Somatization Inventory) [81-84]. The CSSI-24 is a valid and reliable instrument that assesses a variety of nonspecific somatic symptoms [81].

Pain drawings are used to elicit information about the location of pain symptoms [85,86]. A growing body of evidence supports pain drawings as an assessment of pain among children [85].

Fecal samples were collected at baseline and at 8 months for measurement of the intestinal bacterial composition. This is an important background variable that can affect how effective the intervention is for a participant and thus has prognostic significance for the treatment outcome [87].

The Bristol stool chart (BSC) is a well-recognized tool designed to classify the form and consistency of feces into 7 categories [88]. It is used in both clinical and experimental fields [89] and has been used to assess the intestinal transit rate. Normal stool consistency is considered type 3 to type 5 on the BSC [90].

##### Other Somatic Symptoms

Other somatic symptoms are measured with the CSSI-24 [81-83].

Somatic and psychosocial symptoms are also measured with questions from a well-used Swedish survey, Life and Health – Young People (LH-YP) [91]. The questions are in line with the types of questions used in the Health Behavior in School-aged Children study, a cross-national study coordinated by the World Health Organization’s Regional Office for Europe [28].

##### General Health

Self-rated health is measured with a single question: “How do you rate your general health?” The response options range from 1 to 5 (1=very poor, 2=poor, 3=neither good nor poor, 4=good, and 5=very good) [92]. It has been proven to predict mortality and morbidity [93] and to be a valid and reliable item [93,94].

##### Mental Health and Stress

Depressive symptoms are measured with the Children’s Depression Screener (ChilD-S). The ChilD-S is an 8-question validated screening instrument for depressive symptoms designed for children aged 9 to 12 years [95,96]. The recommended cutoff for depression is ≥13 when the child is investigated in psychiatric or psychosomatic care, according to the Swedish translation of the ChilD-S.

The questionnaires also include 8 questions about stress, anxiety, mood, and happiness. These questions are derived from the LH-YP [91].

Stress was also measured with salivary cortisol. Saliva was collected using oral polymer swabs and tubes (Salimetrics LLC). To measure the morning and evening components of the cortisol circadian rhythm, saliva was sampled as soon as possible after awakening while still in bed in the morning and at least 1 hour after last food intake in the evening on a weekday. Collection times were noted. Saliva cortisol has previously been used as a marker of stress for infants, youth, and adults within the research group (eg, with 11- to 12-year-old girls) [97]. The samples were centrifuged and stored at –20 ⁰C in the university hospital in Örebro and then sent on dry ice to the laboratory at the university hospital in Linköping, where they will be analyzed using a commercial enzyme immunoassay method (Salivary Cortisol Enzyme Immunoassay Kit; Salimetrics LLC) [98]. The results will then be sent back to Örebro University Hospital for evaluation.

##### Sleeping Habits

Questions regarding sleeping habits are derived from the LH-YP [91].

##### School Attendance and Function

School attendance and function are measured with 2 questions created by the research group.

##### Physical Activity

A direct measurement of physical activity was obtained using accelerometers with 3 axles (GT3X; ActiGraph) [99]. The participating girls were instructed to wear the accelerometer for 7 days while they were awake.

##### Enjoyment

The validated Physical Activity Enjoyment Scale (PACES) was customized for dance and yoga in this study. PACES was originally developed to measure enjoyment of physical activity and contains 16 items [100,101].

Enjoyment of the intervention was also evaluated with a graphic rating scale [102], which has been proven to have good consistency and stability [103]. The question was “How do you experience dancing/yoga/relaxation while you perform it?” The rating scale ranges from entirely negative to entirely positive.

##### Experiences

To investigate the girls’ experiences of their participation in the intervention and the legal guardians’ experiences of how the girls were influenced by participating in the intervention, qualitative interviews were conducted a few weeks after the intervention ended. The interviews were face-to-face, with the girl and her legal guardian interviewed separately, and semistructured. They were conducted by the same team each year for internal consistency. The interviews were based on open-ended questions from an interview guide, and participants were encouraged to speak freely about their experiences. Prompts (eg, “Could you tell me more?”) were used to obtain richer material.

##### Quality of Life

Quality of life (QOL) is measured with the Kidscreen-10 index [104], which is an instrument with good psychometric features that assesses children’s and adolescents’ subjective health and well-being. It is used in public health and clinical medicine disciplines in multiple countries. An algorithm for mapping the Kidscreen-10 index onto the Child Health Utility Index 9D (CHU9D) utility scores will be used [105].

QOL is also measured with the EuroQol visual analog scale, which represents health status at the moment of evaluation and ranges from 0 (extremely bad) to 100 (excellent quality of life) [106]. Its validity and reliability are reported to be good [107,108].

##### Resource Usage

Both the girls and their legal guardians are asked about resource consumption, such as visits to primary care, school health care, and other open care. These questions were created by the research group.

##### Food Habits

Questions about food habits were derived from the LH-YP [91].

#### Covariates

To assess covariates, we asked about (1) demographics; (2) parental abdominal and gastrointestinal morbidity; (3) the girl’s background, including mode of delivery (caesarean or vaginal), antibiotic consumption early in life, dance and yoga experiences, menarche, etc; and (4) safety at home, in school, during leisure time activities, on social media, and at other places were the girls spend their time.

### Analyses

#### Statistical Analysis

The Just in TIME study follows the intention-to-treat paradigm. Complementary analyses will be performed per protocol and according to the number of sessions the participant attends.

For baseline statistics between the groups, descriptions will be constructed using frequencies and proportions for categorical data and means and standard deviations for continuous variables. Baseline characteristics will be compared using the Fisher exact test for dichotomous outcomes, the Mantel-Haenszel chi-square test for ordered categorical outcomes, and the Mann-Whitney U test or unpaired *t* test for continuous outcomes. A *P* value of <.05 for the 2-tailed test will be considered statistically significant for all outcomes. When deemed necessary, correction for multiple significance will be performed.

The primary outcome analysis will initially be performed using a Fisher exact test to evaluate differences between groups. To analyze the change in scores from baseline between groups for approximately normally distributed variables, a repeated-measures covariance pattern mixed model will be used, adjusting for significant differences at baseline. To identify predictive factors at baseline that might be associated with the primary outcome, a univariable logistic regression analysis followed by a stepwise multiple logistic regression analysis will be performed.

The distribution of continuous variables will be described using the mean and standard deviation or median and interquartile range. Categorical variables will be described with numbers and percentages.

Study participants who choose to leave the intervention before the study is finished are encouraged to complete all assessments. Missing data will be handled with multiple imputations or within the mixed model.

#### Cortisol Analyses

To study the difference between morning and evening values, the evening-morning cortisol quotients will be calculated by dividing each girl’s evening cortisol value by the morning cortisol value. To study potential changes in evening-morning cortisol quotients between baseline and 4 and 8 months, the median change score will be calculated by subtracting baseline cortisol morning samples from 4-month morning cortisol samples and 8-month morning cortisol samples, and the same procedure will be performed for the evening samples.

#### Feces Analyses

Participants received a kit containing everything needed to perform the fecal test at home. The test was stored in the freezer at home for approximately 1 week and was subsequently delivered to the research team in a small freezer bag. The samples are stored at –80 ⁰C at the university hospital in Örebro, and analyses of the intestinal microbiota and short-chain fatty acids will be performed.

#### Analyses of Physical Activity

The frequency of the accelerometer collecting data will be set to 30 Hz. Data will be processed in ActiLife (version 6.13.4; ActiGraph). Raw data from the accelerometers will be converted to 10-second epochs. Valid wear time will be considered to be at least 480 minutes per day, and minimum valid days will be set to 4 days, including 2 weekdays and 1 weekend day. Total counts per day, sedentary time, and activity of moderate or higher intensity will be measured.

#### Qualitative Analyses

The interviews will be transcribed verbatim and analyzed with inductive content analysis, as described by Elo and Kyngäs [109]. The analysis process will initially include getting to know the material, generating codes, and identifying categories. Data will be analyzed using the NVivo software program (QSR International) for qualitative data analysis [110]. All of the authors will discuss the coding process until it is agreed upon and the codes will be grouped into subcategories that reflect the core message of the interviews. Thereafter, the subcategories will be abstracted into generic and main categories [109].

#### Health Economic Analysis

The health economic evaluation will be performed as a cost-utility analysis using individual data [111]. Societal costs (including health care, informal care, and school health care) will be considered. Gained quality-adjusted life-years (QALYs) will be used to measure the effects. QOL will be measured with Kidscreen-10 [104], and an algorithm for mapping the Kidscreen-10 index onto the CHU9D utility scores will be used [105]. Questions about resource use and QOL are asked to both girls and their legal guardians at all follow-ups. Cost-effectiveness ratios will be based on the changes in QALY and net costs for the intervention group compared with the control group. The results will be presented as an incremental cost-effectiveness ratio (ICER), which is expressed as ICER = (Ca – Cb)/(Ea – Eb), where “Ca” is the cost of the intervention, Cb is the cost of the comparator, Ea is the effectiveness of the intervention, and Eb is the effectiveness of the comparator.

### Ethics and Dissemination

The study is conducted in accordance with the standards of Good Clinical Practice and in agreement with the Declaration of Helsinki, and it is approved by the Regional Ethical Review Board in Uppsala, Sweden (No. 2016/082 1-2). Since all participants are younger than 15 years, written informed consent was provided by legal guardians. The information letter included written information about the study, including the purpose and procedures, the voluntary nature of participation, and the option to withdraw at any time. The participants are also guaranteed confidentiality and secured data storage. In addition, all legal guardians and the girls in the study were invited to an information meeting before the start of the study, where verbal explanations of all the procedures were given.

Any adverse events or harm arising from study participation will be reported and managed by the instructors and the research team in accordance with ordinary health care routines. All participants who report 13 or higher on the ChilD-S [95] are offered a clinical evaluation with a psychologist or a psychiatrist and, if needed, are referred to other health care resources, such as the school health service or child and adolescent psychiatry care, without further engagement in the study.

Data are collected in both pen-and-paper and digital formats and securely stored at the University Health Care Research Center. The questionnaire was pretested on an age-appropriate group to determine whether the wording and length of the questionnaire were appropriate for the study’s target group. No unauthorized persons have access to the collected data, either throughout or after the conclusion of the study.

All samples collected in this study are registered in a biobank in Region Örebro County and handled according to the current biobank laws and regulations. The samples are coded to protect the study participants’ identification. All samples and the code list are stored securely and separately to prevent unauthorized persons from having access to them.

### Patient and Public Involvement

A number of girls aged 9 years were involved in composing the questionnaire, selecting research questions, and the developing the intervention. Patients and the public were not involved in the development of the study design, overall measures, recruitment, or conducting of the study. The final results of this study will be disseminated to participants through presentations by the research team at health care and community forums.

## Results

Ethical approval was received in March 2016 and the data collection began in October 2016.

In total, 172 participants were recruited in 3 waves and the intervention was performed in 3 periods from 2016 through 2019. In summary, 121 girls were eligible and allocated. A total of 64 participants were allocated to the intervention group, and 57 participants were allocated to the control group. Data collection of the postintervention follow-ups are ongoing and the final 5-year follow-up will be completed by fall 2023. We expect to publish the first results of the study in the beginning of 2021.

## Discussion

Considering that FAPDs are prevalent among young girls, cost-effective and easily accessible interventions are warranted to reduce the negative consequences arising from these disorders. Physical activity is an effective strategy, but intervention studies are needed to better understand what types of activities facilitate regular participation for this target group. This study will provide new insights regarding the effectiveness of dance and yoga as an active and health-strengthening intervention for girls aged 9 to 13 years with FAPDs. To our knowledge, this is the first study to investigate the influence of these activities combined in an after-school setting.

Worth mentioning is that the current study cannot determine whether one part of the intervention had a greater effect than another. The aim was to evaluate the combination of dance and yoga and the effect of the entire intervention. The supportive group setting, the instructors, the music, or specific parts of the intervention may influence the health effects in different ways. Additionally, it is possible that behavior change alone (ie, engaging in a new organized activity) could contribute to changes in abdominal pain. As always, when testing complex interventions, there are many factors that can affect the outcomes. However, this is also the fact in clinical and real-life situations, and we have chosen a randomized design to account for confounding factors as much as possible.

Methodological strengths of the study include the randomized controlled design, the preregistration in a clinical trials registry, the long follow-up, and the combination of quantitative and qualitative measures. We also verified the FAPD diagnosis with an examination and laboratory evaluation performed by experienced physicians before including the participants in the study.

Several aspects of our trial design are worth noting as potential limitations. The timing of the intervention period followed the Swedish school year, which meant that we conducted the baseline measurement after the summer holiday, when the symptoms from FAPDs can be lower than at other times during the school year. This may have resulted in somewhat inflated exclusion rates, as some girls did not reach the inclusion criteria at the time point for inclusion. Moreover, due to the age of the target group, we need to rely on legal guardians to assist with data collection, which might influence the outcome. There is a possibility that girls with more engaged legal guardians could be overrepresented in the study groups. Intervention logistics, such as avoiding conflicts between the dance and yoga class and the participants’ school curriculum and offering the intervention in an easily accessible studio, are worth considering in future research and in dissemination plans if the activity is being implemented in usual care.

In addition, regarding the biomarker and physiological measures (saliva cortisol, feces, and accelerometer data), we cannot verify how meticulously the participants followed the prescribed guidelines.

The results from this study will broaden the knowledge of how nonpharmacological interventions can be valuable for overcoming the future challenge of reducing the burden of FAPDs for young girls and their families. Combined dance and yoga could be an example of an easy-access joyful intervention with promise as a complementary treatment.

To conclude, this randomized controlled study with 150 participants will investigate the effects of combined dance and yoga in girls with FAPDs. The primary aim is to study the effects of the intervention on abdominal pain, but several other aspects of FAPDs in young girls will also be studied. The results from this intervention study may provide useful information for caregivers in school health care, primary health care, and pediatric outpatient clinics.

## Abbreviations

BSC: Bristol stool chart
CBT: cognitive behavioral therapy
CHEERS: Consolidated Health Economic Evaluation Reporting Standards
ChilD-S: Children’s Depression Screener
CHU9D: Child Health Utility Index 9D
CONSORT: Consolidated Standards of Reporting Trials
COREQ: Consolidated Criteria for Reporting Qualitative Research
CSSI-24: Children’s Somatic Symptoms Inventory-24
FAPD: functional abdominal pain disorder
FGID: functional gastrointestinal disorders
FPS-r: face pain scale-revised
IBS: irritable bowel syndrome
ICER: incremental cost-effectiveness ratio
LH-YP: Life and Health – Young People
PACES: Physical Activity Enjoyment Scale
QALY: quality-adjusted life-years
QOL: quality of life
SPIRIT: Standard Protocol Items: Recommendations for Interventional Trials
TGA-IgA: immunoglobulin A antibodies against type 2 (tissue) transglutaminase
TIDieR: Template for Intervention Description and Replication
